# Internet Searching About Disease Elicits a Positive Perception of Own Health When Severity of Illness Is High: A Longitudinal Questionnaire Study

**DOI:** 10.2196/jmir.5140

**Published:** 2016-03-04

**Authors:** Kai Sassenberg, Hannah Greving

**Affiliations:** ^1^ Leibniz-Institut für Wissensmedien Social Processes Lab Tübingen Germany; ^2^ Faculty of Science University of Tübingen Tübingen Germany

**Keywords:** health information, Internet search, threat, perception of own health, chronic inflammatory bowel disease

## Abstract

**Background:**

The Internet is one of the primary sources for health information. However, in research, the effects of Internet use on the perception of one’s own health have not received much attention so far.

**Objective:**

This study tested how Internet use for acquiring health information and severity of illness influence patients with a chronic disease with regard to the perception of their own health. Negative psychological states are known to lead to preferential processing of positive information. In particular, the self-directed nature of Internet use provides room for such biases. Therefore, we predicted that patients experiencing negative health states more frequently, due to more frequent episodes of a chronic illness, will gain a more positive perception of their health if they use the Internet frequently to gain health information, but not if they use the Internet rarely. This effect was not expected for other sources of information.

**Methods:**

A longitudinal questionnaire study with two measurement points—with a 7-month time lag—tested the hypothesis in a sample of patients with chronic inflammatory bowel disease (n=208). This study assessed patients’ frequency of Internet use, their participation in online social support groups, their use of other sources of health information, and several indicators of the participants’ perceptions of their own health. A structure equation model (SEM) was used to test the predictions separately for Internet searches and other sources of information.

**Results:**

Data analysis supported the prediction; the interaction between frequency of health-related information searches and frequency of episodes at the first measurement point (T1) was related to participants’ positive perceptions of their own health at the second measurement point (T2) (B=.10, SE=.04, *P*=.02) above and beyond the perceptions of their own health at T1. When participants used the Internet relatively rarely (-1 SD), there was no relationship between frequency of episodes and positive perceptions of their own health (B=-.11, SE=.14, *t*_203_=-0.82, *P*=.41). In contrast, when participants used the Internet relatively often (+1 SD), the more frequently they had those episodes the more positive were the perceptions of their own health (B=.36, SE=.15, *t*_203_=2.43, *P*=.02). Additional SEM analyses revealed that this effect occurs exclusively when information is searched for on the Internet, but not when other sources of information are consulted, nor when online social support groups are joined.

**Conclusions:**

The results of this study suggest that patients might process information from the Internet selectively, in an unbalanced, biased fashion, with the formation of a self-serving (ie, positive) perception of own health. At the same time, this bias contributes to the ability of patients to cope psychologically with their disease.

## Introduction

### Background

The Internet provides lay people with access to health information that was in earlier days available only to physicians and other health care professionals. Already in 2001, about 40% of Internet users searched online for health information [1]. Meanwhile, Internet use in the health domain has become so popular that, based solely on search engine query data, influenza epidemics can be detected [2]. Access to health information through the Internet has the potential to create better-informed patients and to enable them to become engaged in caring for their health; at the same time, health information on the Internet is often inaccurate or incomplete [3-5].

The risks and benefits associated with the availability of health information on the Internet do not result only from the content of this information, but also from how patients process it (see Kalichman et al [6]). Current research aims to test the impact of severity of illness, as a factor that is likely to affect information processing, and the frequency of Internet use for health information acquisition on the perception of one’s own health.

### A Preference for Positive Over Negative Information

Health information is processed in a biased fashion. Positive or self-serving information (eg, suggesting own good health state) is, for instance, more easily accepted and less avoided than negative self-relevant information (eg, suggesting own illness) [7]. Preferential processing of self-serving information has been demonstrated at different stages of information processing, such as attention, encoding, and recall; for an overview, see De Hoog et al [8] and Shepperd et al [9]. Other results suggest that individuals have a strong tendency to prefer positive over negative information about their own health [10].

There are, however, reasons to assume that the extent to which the positive, or self-serving, bias occurs depends crucially on individuals’ health states and their psychological states resulting from them [10,11]. Research across domains has revealed a stronger positive bias in individuals in a negative state (ie, a state experienced as aversive, such as feeling uncertain, threatened, or bad), which is likely experienced by ill individuals. To be more precise, studies have shown that in negative psychological states as compared to positive states (ie, states experienced as enjoyable or neutral states), positive information receives more attention than negative information. This is particularly true when individuals are focusing on losses rather than gains [12], when they are experiencing negative rather than positive emotions [13], or when they are reminded of negative rather than positive experiences [14,15]. In addition, experiencing low control over one’s current and future situation (ie, another negative state) elicits a positive bias [16,17]. Moreover, being in a negative state does not only lead to a positive bias regarding attention, but also regarding decision-making [18]. This process is called counter-regulation [19] because a negative state is counteracted by attention to positive stimuli [12]. Taken together, negative psychological states that are likely to come along with periods of illness have been shown to result in a positive bias in information processing.

We believe that during Internet searches, a positive bias in information processing is very likely to occur because searching and surfing the Internet is completely self-directed (ie, not guided by external restrictions). Also, it can be done via multiple paths due to the hypertext structure and the virtually unlimited amount of information available [20]. Other sources of health information usually provide participants with more guidance. In social interactions, doctors or other health professionals communicate information to patients based on their own aims; ideally, they provide patients with balanced and unbiased information to empower them to autonomously make informed decisions [21]. Even on television, in newspapers, or in books (ie, noninteractive sources), information about an illness is usually designed in a way such that readers get a certain unbiased set of information (eg, Anderson and Nottingham [22]). In comparison, information acquisition on the Internet is completely self-directed because users can stop reading a text and “surf on” at any given point in time. This is due to the large quantity and the heterogeneous mass of information with regard to content being available online. Therefore, when the available information allows for several interpretations and a self-directed information search [23,24], as is the case on the Internet, negative psychological states are more likely to guide information processing (ie, influence the behavioral steps of the Internet search process) toward preferential processing of positive information. This is due to the fact that internal negative and positive states can only exert influence on information processing if the search process is self-directed, as it is during Internet searches, but not if the information search is externally guided, as in the case of other sources. In other words, the Internet provides optimal degrees of contextual freedom, which Rothermund [19] named to be the prime precondition for the occurrence of counter-regulation.

Indeed, experimental research [25,26] has shown that a negative psychological state influences Internet search behavior. In a series of experiments with healthy participants, threat was induced based on either providing participants with an ostensible diagnosis [7] or asking them to think and write about a threat they were currently experiencing. Participants then searched for information in a health domain (ie, either information related to the diagnosis or about living organ donation). Under threat, compared to a no-threat control condition, more positive search terms were generated, more positive links were selected from a link list, more positive information was recalled, and the search topic was evaluated as more positive. Positive search terms, links, and information—in the case of searches about living organ donation— focused, for example, on the fact that organ donations give a “second life” to patients or on the circumstances under which donated organs are in good condition. These studies suggest that patients using the Internet to acquire information about their own illness and health will likewise apply a positive bias, because patients’ actual health states potentially induce negative psychological states. Patients with a chronic illness—a group that frequently uses the Internet [27]—will, for instance, suffer not only physically but also feel bad (ie, experience a negative psychological state) when they go through an episode of their illness. This, in turn, forms the basis for a positive bias during health-related Internet searches [25,26].

What are the long-term consequences of such a positive bias during Internet searches? As mentioned above, research on the immediate outcomes of Internet searches has shown that negative states lead to better memory for positive information and more positive attitudes toward the target around which the search centers [25,26]. Therefore, in the long run, the perception of the illness and a patient's own health should become more positive the more often Internet searches are conducted by individuals in a negative psychological state. Hence, we predict that patients with chronic illnesses, who use the Internet frequently to search for health-related information, will have a more positive perception of their health the more frequently they experience episodes of their illness. In contrast, this relationship between the frequency of episodes and positive perception of their own health will not occur in patients who rarely use the Internet to search for health-related information.

## Methods

### Overview and Study Design

A longitudinal study with two measurement points and a time lag of 7 months was conducted with patients who suffer from chronic inflammatory bowel disease to test this hypothesis. We focused on this chronic disease as it is characterized by infrequently occurring acute episodes of illness, which substantially restrict patients in their ability to cope with everyday demands; the episodes are, thus, likely to elicit negative psychological states. Moreover, patients with chronic diseases rely particularly on the Internet as an informational source because their illnesses strongly restrict them in their daily and social activities, which is why they are often unable to leave the house [27]. This also applies to patients with inflammatory bowel disease. These patients, thus, formed an appropriate group of patients for our study.

We used three indicators for the positive perception of own health that captured the immediate outcome of the positive bias—the perception of the risks resulting from the disease—as well as how it affects participants personally—health-related stress and health-related self-esteem. As information about their health and their disease is self-relevant to patients, we expected that our hypothesis would apply to all three indicators of perception of own health.

Participants were recruited and data were collected via the Internet in order to gain a sample of patients who were experienced in using the Internet for health-related purposes. To be able to test whether the predicted frequency of Internet searches by frequency of episodes interaction occurs for all sources of information or just for health-related Internet searches, we assessed online social support group participation and the consultation of offline sources of health-related information (ie, interactive and noninteractive sources). No frequency of episodes by source of information interaction was expected for sources other than Internet searches because these other sources do not provide the degree of self-directedness required to provide room for counter-regulation and the preferential processing of positive cues.

### Participants

Patients with chronic inflammatory bowel disease participated in an open, online questionnaire study with two measurement points that were 7 months apart. Participants were recruited via the German Association for Crohn’s Disease and Ulcerative Colitis, which has more than 20,000 members and is, thus, the biggest organization of patients with chronic inflammatory bowel disease in German-speaking countries. The association advertised the study in their members’ journal, on their website, via email, and on social networking sites.

When following the link in the advertisement, participants first had to provide informed consent online. After receiving information about the duration, the content, and the aims of the study, as well as the data storage policy, each participant had to actively check a box and, thereby, indicate informed consent. Participation was completely voluntary. To get access to the survey, participants had to enter their email address into a Web form. Email addresses were stored separately from the other data and were only accessible to the system administrator (ie, not to the researchers), who deleted them after the second wave of data collection was completed. Participants received an email with a link to the actual questionnaire.

The reported study was ethically approved by the ethics committee of the Faculty of Medicine at the University of Tübingen, Germany. As compensation, participants who completed questionnaires from both waves received a gift voucher of €10—approximately US $11—to be redeemed at an online sales platform.

### Main Questionnaire

All measures of the survey at the first measurement point (T1) and the second measurement point (T2) were assessed via the online survey program, Questback. Items were presented in a fixed order. First, participants answered demographic questions (ie, gender, age, and education) and general questions about their health and illness (ie, type of diagnosis, time since receipt of diagnosis, time since occurrence of symptoms, number of episodes, current acute episode, and severity of acute episode). These questions included the assessment of the key predictor, frequency of episodes. Within a larger battery of measures, participants’ health-related self-esteem, health-related stress, and health-related risk perceptions were assessed and served as the key dependent measures. Finally, participants answered questions about their Internet use—the second key predictor—the Internet services they used, which online social support group they joined, and which offline sources of information they used. The functionality and readability of the questionnaire was pretested by healthy participants. The questionnaire contained up to 14 items. Returning to already completed pages of the questionnaire was not possible. It was mandatory to complete the items assessing key concepts. Participants were contacted via email about 7 months after they had completed the questionnaire from the first wave. Those who did not reply within a week received a reminder. All measures at T2 were exactly the same as at T1.

### Measures

*Frequency of episodes* was assessed with an item asking participants to indicate how many acute episodes of illness they had had during the last year. Participants reported a mean of 1.93 episodes (SD 2.10). This indicator was *Z*-standardized for all analyses reported below because the raw values were skewed, as is often the case for frequency counts.

*Health-related information searches on the Internet* was assessed with an item that requested participants to report the frequency of their Internet use for this purpose. Participants provided the answer on a 7-point scale with the following options: *rarely or never* (1), *two to six times per year* (2), *one to two times per month* (3), *one time per week* (4), *two to five times per week* (5), *one time per day* (6), and *several times per day* (7). This item served as a measure of health-related information searches on the Internet.

In the next passage, questions on the use of health information sources had to be answered by checking or not checking boxes. Possible information sources were search engines, forums, encyclopedias, patient association websites, Internet portals, scientific search engines, newsletters, and social networks.

*Online social support group participation* was also assessed by one item—“How often do you visit online social support groups?”—which participants answered again on the 7-point scale used for health-related information searches.

For *other sources of health information,* participants indicated by checking or not checking a box whether they made use of the following offline sources of health information: doctors, family and friends, psychologists or advisory centers, books, presentations, newspapers and journals, and television or radio. We created two indices from these answers by counting the number of checked boxes, separately for social interactive sources (ie, doctor, friends and family, and psychologists or advisory centers) and noninteractive sources (ie, books, presentations, newspapers and journals, and television or radio).

*The positive perception of own health* was, as mentioned above, captured by using three different indicators: health-related self-esteem, health-related stress (reversed), and health-related risk perception (reversed). Health-related self-esteem was measured by five items adopted from the social state self-esteem subscale of the State Self-Esteem Scale [28]: “Due to my chronic illness, I feel self-conscious,” ”Due to my chronic illness, I feel displeased with myself,” “Due to my chronic illness, I feel inferior to others at this moment,” “Due to my chronic illness, I am worried about what other people think of me,” and “Due to my chronic illness, I feel concerned about the impression I am making.” All of these items were reverse coded as in the original scale (T1 alpha=.86, T2 alpha=.87). The health-related stress that participants experienced due to their illness was assessed by seven items adopted from different stress appraisal measures [29,30]; for example, “I feel totally helpless with my chronic illness,” “I feel that my chronic illness is beyond my control,” and “My chronic illness impacts me greatly” (T1 alpha=.88, T2 alpha=.91). Finally, participants’ health-related risk perception in relation to their disease was measured with five self-developed items. We developed these items because, to the best of our knowledge, no available scale captures the perceived risks associated with inflammatory bowel diseases: “Inflammatory bowel diseases may result in bowel cancer after a long time,” “Inflammatory bowel diseases greatly restrict social life,” “Inflammatory bowel diseases often come along with intestinal incontinence,” “Inflammatory bowel diseases make you feel constantly ill,” and “Inflammatory bowel diseases come along with many adverse effects” (T1 alpha=.54, T2 alpha=.56). The internal consistencies of this scale were not as high as one would have wished, which could unfortunately not be substantially improved by excluding single items. As the scale was approximately normally distributed, we chose to average the items into a single index. Ratings for all three scales were on a 5-point Likert scale ranging from 1 (*does not apply at all*) to 5 (*completely applies*).

### Inclusion and Exclusion Criteria

Participants had to be 18 years of age or older and have chronic inflammatory bowel disease. The only exclusion criterion was that the answers to the two main disease-related variables—time since receipt of diagnosis and number of episodes per year—had to be within a plausible range: < 60 years and < 20 episodes, respectively.

### Data Analysis

**Figure 1:**
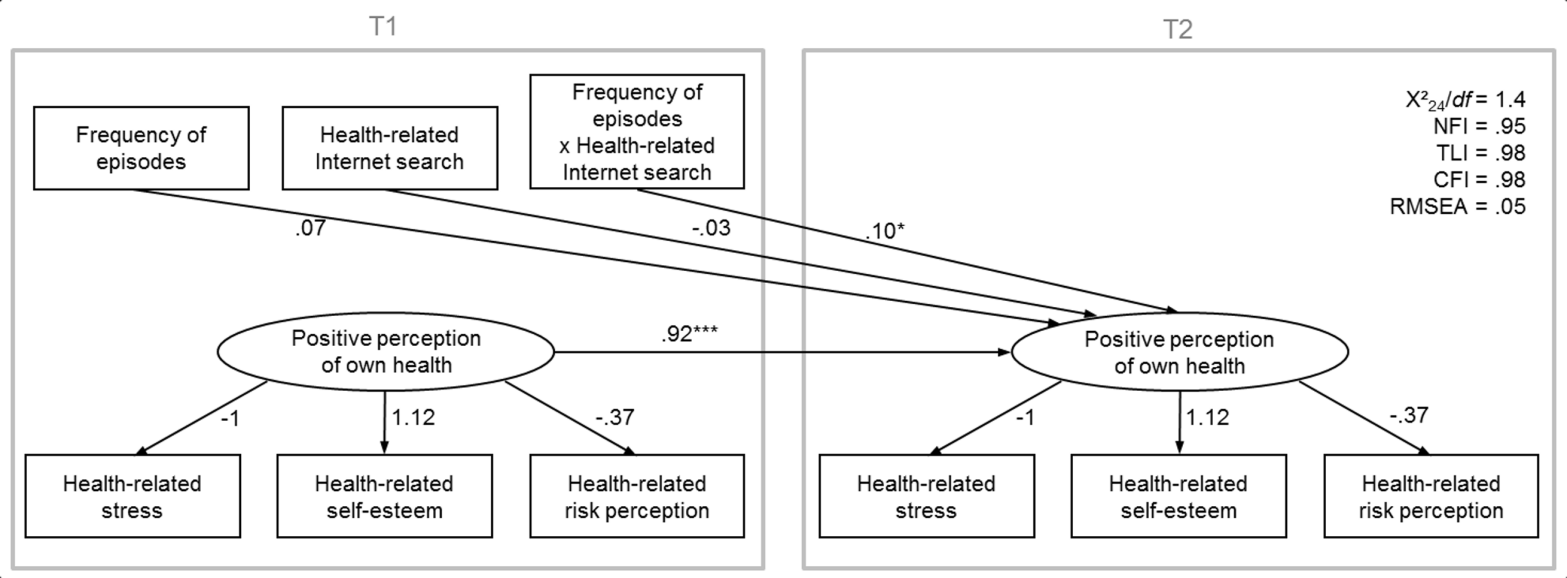
Structure equation model (SEM) for the effects (unstandardized regression coefficients, B) of frequency of episodes, frequency of health-related information searches, and their interaction from the first measurement point (T1) on the latent variable, positive perception of own health, and from the second measurement point (T2), controlling for positive perception of own health from T1. NFI: normed fit index; TLI: Tucker-Lewis index; CFI: comparative fit index; RMSEA: root mean square error of approximation; ns: not significant; *P<.05; ***P<.001.

To test the hypothesis, a structure equation model (SEM) was applied using SPSS Amos version 22 (IBM Corp). In this model, the three manifest indicators of the positive perception of own health were summarized as a latent variable, separately for T1 and T2. Relationships between the assessed indicators and the latent variables were set equally across both time points. The model regressed the perception of own health at T2 on the same variable at T1, as well as on frequency of health-related information searches, frequency of episodes, and their interaction (see Figure 1). Before the interaction was computed, both variables were *Z*-standardized.

The correlation between the measurement error of frequency of episodes at T1 and the error term for positive perception of own health at T1 was set free as it was likely that both were related. Moreover, measurement errors of health-related stress, health-related self-esteem, and health-related risk perception at T1 were allowed to covary with their respective measurement errors at T2. This was done because we assumed that components of these indicators that are not part of the latent variable of positive perception of own health might still be stable. The model did not include any further covariates. To test for the opposite causal relationship, we also computed a model in which the perception of own health at T1 predicts the interaction between frequency of episodes and health-related Internet searches at T2.

Finally, additional models were tested to find out whether Internet searches alone or also other types of information searches interact with the frequency of episodes in their impact on the perception of own health. These alternative models replaced health-related information searches on the Internet by social interactive sources, by noninteractive sources, or by participation in online social support groups. Except for this change in the predictor and its interaction with frequency of episodes, the alternative models did not exhibit any changes in comparison to the main model. We did not expect to find an interaction between the respective information sources and the frequency of episodes on positive perception of own health in these models.

## Results

### Sample Description

A total of 8 out of 216 participants (3.7%) who completed both questionnaires were excluded from the analyses reported below based on the exclusion criteria: time since receipt of the diagnosis (> 60 years) and number of episodes during the last year (> 20 episodes). Thus, 208 participants were included in the analyses: 154 women (74.0%), 54 men (26.0%), mean age 37.77 years (SD 11.51, range 18-63). Out of these participants, 0.5% (1/208) had not finished school, 8.7% (18/208) had a certificate of secondary education after 9 years of schooling (German: Hauptschulabschluss), 26.4% (55/208) had a general certificate of secondary education after 10 years of schooling (German: Mittlere Reife), 30.8% (64/208) had a high school degree (ie, a certificate of general university maturity after 12 years of schooling), 10.1% (21/208) had a polytechnic degree, and 23.6% (49/208) had a university degree. Thus, the sample was well educated.

The self-report about participants’ diseases revealed that 57.7% (120/208) of the participants had Crohn’s disease, 40.4% (84/208) had ulcerative colitis, 1.0% (2/208) had indeterminate colitis, and 1.0% (2/208) reported to have a disease different from the aforementioned ones. The participants received the diagnosis an average of 11.79 years (SD 10.01) ago and had had symptoms for an average of 13.61 years (SD 10.83).

Participants reported using the following online sources of health information: search engines (196/205, 95.6%), forums (154/205, 75.1%), encyclopedias (109/205, 53.2%), patient association websites (108/205, 52.7%), Internet portals (68/205, 33.2%), scientific search engines (eg, Google Scholar and PubMed) (53/205, 25.9%), newsletters (51/205, 24.9%), and social networks (44/205, 21.5%). Out of 208 participants, 3 (1.4%) did not answer this question. In addition, they indicated using the following offline sources of health information: doctors (187/204, 91.7%), family and friends (108/204, 52.9%), psychologists or advisory centers (52/204, 25.5%), books (131/204, 64.2%), presentations (113/204, 55.4%), newspapers and journals (90/204, 44.1%), and television or radio (67/204, 32.8%). Out of 208 participants, 4 (1.9%) did not answer this question.

### Dropout Analysis

The first page of the questionnaire had 319 hits. A total of 258 patients with inflammatory bowel disease completed the whole questionnaire at T1 (258/319, 80.9%) and 216 patients completed the questionnaire at T2 (216/319, 67.7%): dropout rate of 16.3% (42/258) from T1 to T2. These subsamples did not differ with respect to the following variables: gender (χ^2^_1_=0.1, *P*=.85), type of diagnosis (χ^2^_3_=1.5, *P*=.67), age, time passed since receipt of diagnosis, time since occurrence of first symptoms, and number of episodes during the last year (*t*<1.5, *P*>.13). Those participants who dropped out of the study used the Internet slightly more often for health-related purposes (mean 4.70, SD 1.32) than the participants who completed the questionnaire at both measurement points (mean 4.25, SD 1.47; *t*_258_=1.84, *P*=.07). As the dropout rate was very small and as we found only one marginal deviation between both subsamples, we considered the remaining sample as suitable for testing our prediction.

### Basic Analysis

The correlations between variables and their means and standard deviations are displayed in Table 1.

**Table 1:** Correlation of all variables.

Number	Variables	Mean	SD	1	2	3	4	5	6	7	8	9
1	Health-related Internet search T1^a^	4.24	1.46									
2	Frequency of episodes T1	1.95	2.11	.07								
3	Health-related stress T1	2.63	0.95	.19^b^	.22^b^							
4	Health-related self-esteem T1	3.42	1.06	-.16^c^	-.23^b^	-.66^d^						
5	Health-related risk perception T1	3.30	0.65	.17^c^	.20^b^	.38^d^	-.30^d^					
6	Health-related Internet search T2^e^	4.07	1.48	.61^d^	.04	-.01	.05	.08				
7	Frequency of episodes T2	1.64	1.98	.16^c^	.53^d^	.23^b^	-.16^c^	.21^b^	.04			
8	Health-related stress T2	2.58	0.99	.18^b^	.12	.75^d^	-.55^d^	.26^d^	.08	.25^d^		
9	Health-related self-esteem T2	3.49	1.08	-.19^b^	-.15^c^	-.62^d^	.72^d^	-.32^d^	-.07	-.17^c^	-.73^d^	
10	Health-related risk perception T2	3.17	0.66	.10	.18^b^	.32^d^	-.28^d^	.57^d^	.19^b^	.14^c^	.35^d^	-.40^d^

^a^T1: first measurement point.

^b^ *P*<.05.

^c^ *P*<.01.

^d^ *P*<.001.

^e^T2: second measurement point.

### Test of Predictions

We predicted that frequency of episodes and health-related information searches on the Internet interact in their impact on patients’ positive perceptions of their own health. To be more precise, we expected that in patients using the Internet frequently, but not in patients using it rarely, more frequent episodes should lead to a more positive perception of their own health. Multi-colinearity is often an issue when computing interactions between variables assessed in one source. Yet, our predictors—frequency of episodes and frequency of health-related information searches—were not correlated (r=.07, n=208, *P*=.33).

The predicted model showed a good fit to the data (see Figure 1)—χ^2^_24_=35.0, *P*=.07; normed fit index (NFI)=.954; Tucker-Lewis index (TLI)=.977; comparative fit index (CFI)=.985; root mean square error of approximation (RMSEA)=.046. When the measurement errors at T1 were not allowed to correlate, the model nevertheless showed a satisfactory, though not excellent, fit to the data—χ^2^_23_=49.7, *P*=.001; NFI=.934; TLI=.942; CFI=.963; RMSEA=.074.

**Figure 2:**
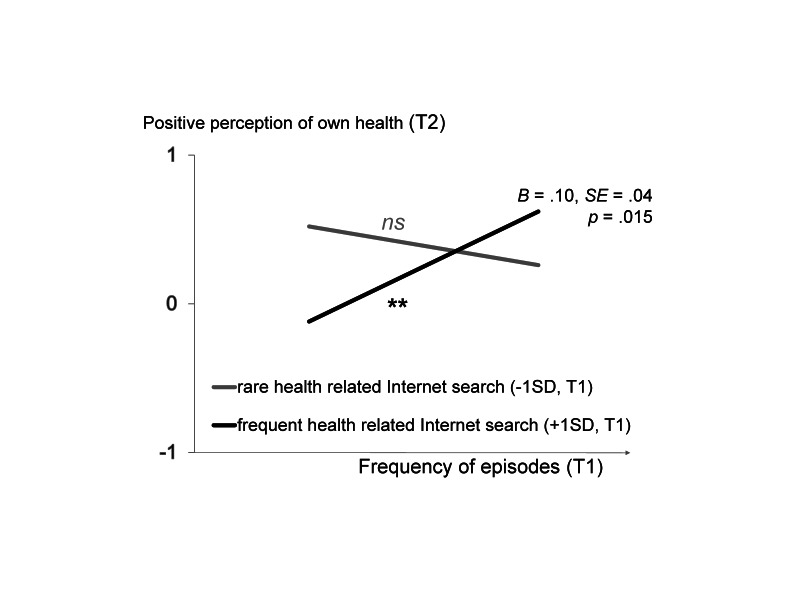
Results from the regression analysis within the structure equation model (SEM) analysis with the latent variable, positive perception of own health, from the second measurement point (T2) as criterion, and frequency of episodes and frequency of health-related information search as predictors controlling for positive perception of own health from the first measurement point (T1). ns: not significant; **P<.01.

Frequency of health-related information searches at T1 did not predict the perception of own health at T2 (B=-.03, SE=.04, *P*=.50), whereas there was a trend for an effect of frequency of episodes at T1 on perception of own health at T2 (B=.07, SE=.04, *P*=.07). More importantly, we found the predicted interaction between frequency of health-related information searches and frequency of episodes at T1 on positive perception of own health at T2 (B=.10, SE=.04, *P*=.02). As depicted in Figure 2, when participants used the Internet relatively rarely (-1 SD) to search for health-related information, there was no relationship between frequency of episodes and positive perception of own health (B=-.11, SE=.14; *t*_203_=-0.82, *P*=.41). In contrast, when participants used the Internet relatively often (+1 SD) to search for health-related information, the more frequently they experienced episodes the more positive was their perception of their own health (B=.36, SE=.15; *t*_203_=2.43, *P*=.02).

Additional SEMs were computed to gain further insights about this effect. First, a model testing the opposite causal direction did not find evidence for any influence of perception of own health at T1 on the interaction between frequency of episodes and health-related Internet searches at T2 (B=-.03, SE=.10, *P*=.74). This finding provides evidence that the causal direction we assume is underlying the data rather than the opposite causal direction. That is, the perception of own health is affected by the frequency of episodes and the frequency of health-related Internet searches rather than the other way around.

Moreover, another model tested whether the same interaction effect occurred for participation in online social support groups, instead of health-related Internet searches. This analysis served to rule out that any health-related Internet search, which might similarly occur in online social support groups, results in the predicted effects. In this model, no interaction between online social support group participation and frequency of episodes on the perception of own health was found (B=.02, SE=.05, *P*=.63). Hence, in line with our reasoning, the results seem to be driven by Internet searches to acquire health-related information than by Internet use to gain social support.

Finally, two additional models tested whether offline sources of health-related information, namely, social interactive and noninteractive (ie, purely informational) sources, lead to effects similar to those of health-related Internet searches. Again, no interaction occurred between the frequency of episodes and the consultation of health-related offline sources (social interactive sources: B=-.02, SE=.04, *P*=.67; noninteractive sources: B=-.06, SE=.05, *P*=.24). These findings support our expectation that the positively biased perception of own health in case of severe illness refers exclusively to health-related Internet searches.

In sum, the results support the hypothesis that health-related Internet searches for knowledge acquisition and the frequency of episodes associated with the illness have an impact on participants’ perception of own health over time; with increasing frequency of episodes, the perception of their own health became more positive when participants used the Internet for health-related information acquisition often, but not when they used it rarely. The additional analyses did not find similar effects for information acquisition from offline media or other individuals, nor from online social support groups. This indicates that the effects described here are unique for self-directed information acquisition on the Internet.

## Discussion

### Principal Findings

The current research aimed to test the long-term effects of patients’ Internet use on the perception of their own health. We predicted that patients experiencing episodes of illness more frequently have a more positive perception of their health when frequently using the Internet, but not when rarely using the Internet to search for health-related information. This prediction relied on research on counter-regulation [12,13,15,18]. Due to its longitudinal nature and due to the patient sample, the research in this study goes beyond earlier research that had demonstrated among individuals in a negative affective state a positive bias in different stages of the Internet search process (ie, under threat) [25,26].

The interaction between frequency of health-related Internet searches and frequency of episodes of their chronic disease predicted patients’ perception of their own health across a 7-month period. Additional analyses did not find evidence for similar effects for other sources (ie, external sources and social support group participation) or for the opposite causal direction. The results clearly indicated that the effects occur, in line with our prediction, only as a consequence of information-related Internet searches and not when social information sources (eg, friends or health professionals) or other offline information sources (eg, books) were resorted to. This suggests that, in line with our assumption, the self-directed nature of information acquisition on the Internet provides the contextual basis for counter-regulation and its impact on information processing and acquisition. In other words, the degrees of freedom the Internet provides during information searches allow for selective information acquisition and the formation of a self-serving (ie, positive) perception of own health when experiencing episodes of illness more frequently. This interpretation of the findings is speculative insofar as this study did not assess any indicators of this assumed process (ie, room for self-regulation leading to selective processing of information in threatened individuals). At the same time, earlier studies have provided ample evidence that self-regulated information searches on the Internet lead to counter-regulation and a preferential processing of positive information in individuals in a negative state [25,26]. Therefore, it seems justified to conclude that the degrees of freedom users have during Internet searches caused the specific outcome of Internet searches, which is different from outcomes involving resorting to other information sources. Nonetheless, it is worthwhile to explore further in future research the processes underlying the long-term effects of information searches on the Internet that were found here for the first time.

### Comparison With Earlier Work and Strengths

To the best of our knowledge, this study was the first to study long-term effects of frequency of episodes and Internet searches to acquire health-related information. Earlier research on health within the context of the Internet mostly focused on online prevention programs [31,32] and the effects of online social support [33,34].

Beyond its originality, the strengths of this study are its longitudinal design and the fact that several indicators for the dependent variable have been assessed targeting the perception of own health, as well as the perception of the illnesses patients are suffering from. Separate tests for each of the three indicators show the same results as the analysis across indicators reported above.

### Limitations

Only patients with specific chronic diseases were included in the study. In this study, relying on patients with chronic inflammatory bowel diseases allowed for the assessment of a more homogenous and less subjective indicator of the severity of illness, namely, the number of episodes during the last year. In this vein, focusing on one particular group of diseases allowed for the assessment of an appropriate indicator of severity of illness. Nonetheless, further research should definitely aim to replicate the current findings with other chronic diseases and beyond.

One might consider the limited internal consistency of the indicator of health-related risk perception as a weakness of this study. As no scale for risk perception in the context of chronic inflammatory bowel diseases exists in the literature, we developed our own scale that did not work out perfectly. However, given that we use a latent variable for the perception of one’s own health, the error variance included in the risk perception scale does not affect our main statistical test. In addition, risk perception loads relatively low on the latent variable; the model also holds when only the other two indicators of perception of one’s own health are used. For these reasons, we do not consider the unsatisfying internal consistency of the risk perception scale as a threat to the validity of these findings.

A final limitation of this study is that we did not assess what type of information participants studied on the Internet. This would have allowed us to find out whether they select, receive, or remember information in an unbalanced fashion. However, collecting that information and analyzing it across a 7-month period hardly seems feasible. In addition, earlier experimental research has already addressed this aspect and found that all steps of the information search process on the Internet are guided by a preference for positive information in a negative psychological state (ie, threat) [25,26].

### Implications

The findings from this study have ambivalent implications for patients. On the one hand, Internet use seems to help patients with severe illnesses to develop a positive perception of their own health because the information-related Internet searches lead to a more positive picture of their own health situation—in particular, if they are severely affected by their disease (ie, frequently suffer from it). Hence, this research has added one more aspect to the list of positive implications of health information searches on the Internet, such as increased empowerment and heightened compliance among patients using the Internet [35,36]. On the other hand, the positive perception of own health can also be seen as evidence for a bias in information processing on the Internet. As has been found in earlier research on counter-regulation, those in a negative state are more likely to preferably process positive information [12,13,15,18]. In the case of patients’ Internet searches, this might imply that when frequently searching the Internet for health-related information, those in a negative state in particular have a distorted picture about their own health—they might perceive their own situation as far too positive (eg, underestimate risks).

This distorted and possibly too-positive perception, in turn, can have implications for the doctor-patient relationship and the medical treatment of those patients. Internet-informed patients might be less willing to accept their doctors' advice and claim more autonomy in health decisions, due to an increased positive perception of their own health as was found in this study. This might in turn lead to less willingness to comply. Therefore, physicians should pay particular attention to this implication in order to prevent their patients, fatally enough, from making decisions against appropriate treatments. At the same time, patients should be aware of the need to carefully conduct Internet searches and examine the integrity of an information source before relying on its information. Further research is needed to gain more insight about these implications as they rely more on speculative conclusions from this study's findings than on the findings themselves.

### Conclusions

This study provided evidence for long-term outcomes of Internet searches for health information. The more severe the patients’ illnesses (ie, the more frequently they suffer from them), the more that frequent Internet searches lead to a positive perception of own health. Thus, the accessibility of health-related information on the Internet renders patients more informed than in the past when health-related information was accessible only to health professionals; it also opens an avenue for unbalanced information processing. This unbalanced processing might help patients cope emotionally with their situation; however, it might result in a biased perception of their own health, particularly in those who suffer severely from a disease.

## Abbreviations

CFI: comparative fit index
NFI: normed fit index
ns: not significant
RMSEA: root mean square error of approximation
SEM: structure equation model
T1: first measurement point
T2: second measurement point
TLI: Tucker-Lewis index
